# Supplementary material for: Photonics of Some Monomethine Cyanine Dyes in Solutions and in Complexes with Biomolecules
Source: Int J Mol Sci. 2023 Sep 11;24(18):13954. doi: 10.3390/ijms241813954 (PMC10531095; doi:10.3390/ijms241813954)
Supplement: Supplementary file 1 [file ijms-24-13954-s001.zip › ijms-2577122-supplementary.pdf]

Article

Photonics of Some Monomethine Cyanine Dyes in Solutions and in Complexes with Biomolecules

Pavel G. Pronkin and Alexander S. Tatikolov *

N.M. Emanuel Institute of Biochemical Physics, Russian Academy of Sciences, 4 Kosygin Str., 119334 Moscow, Russia; pronkinp@gmail.com

* Correspondence: tatikolov@mail.ru; Tel.: +7-(495)-939-71-71

1. Spectral-Fluorescent Titration

In experiments on spectral-fluorescence titration, MCD **1**–**4** were dosed from their concentrated stock solutions in EtOH. Microvolumes (~0.5–5 μL) were dosed into the measuring cell (dilution 1:500 – 1:1000). In experiments with biomacromolecules, microvolumes of concentrated stock solutions of dsDNA, ssDNA and HSA were injected directly into the cells. Spectral-fluorescent characteristics of MCD in complexes with biomacromolecules were studied by titration of dye solutions (c_{dye} ~ 1 – 2 × 10^{-6} mol L^{-1}) with increasing concentrations of biopolymers. Measurements of the intrinsic fluorescence of HSA in the presence of MCD were carried out at a constant concentration of HSA (c_{HSA} ~ 2.0 × 10^{-5} mol L^{-1}) by titration with increasing concentrations of dyes. After stirring, the absorption and fluorescence spectra of the dyes were recorded several times. Upon registration, the fluorescence and fluorescence excitation spectra were corrected for the spectral characteristic of the excitation channel and for the absorption of the solution.

2. Fluorescence Quantum Yields

Determination of the fluorescence quantum yields of MCD **1**–**4** was performed using the standard relationship:

$$\Phi_{\text{fl}} = \frac{(1-T_{\text{st}})F}{(1-T)F_{\text{st}}}\frac{n^2}{n_{\text{st}}^2}\Phi_{\text{flst}} \approx \frac{F}{F_{\text{st}}}\frac{n^2}{n_{\text{st}}^2}\Phi_{\text{flst}}, \quad \text{(S1)}$$

where $T_{st.}$ and T are the transmission coefficients at the excitation wavelength of the standard and dye solutions, $F_{st.}$ and F are the integrals of the emission bands, and $n_{st.}$ and n are the refractive indices of the standard and dye solutions. Under the condition of equivalent absorbances of the sample and the standard, we may take $T \approx T_{st}$. The experimental error in determining Φ_{fl} was ±10% [S1, S2].

3. Method of Spectral Moments

$$S_l = \int \nu^l \rho(\nu)\, d\nu, \quad \text{(S2)}$$

$$M_l = \frac{1}{s_0}\int \nu^l (\nu - \nu_1)^l \rho(\nu)\, d\nu \quad \text{(S3)}$$

where S_l and M_l are the initial and central spectral moments, l is the order of the moment (l = 1, 2, etc.); $\rho(\nu) = \varepsilon(\nu)/\nu$ is the distribution of the absorption intensity in the spectra; $\varepsilon(\nu)$ is the absorption of the sample, ν is the wavenumber (cm^{-1}), $\nu_1 = S_1/S_0$ is the first moment of the normalized photon distribution, $M^{-1} = 10^7/\nu_1$ is the average position of the band in the wavelength scale [S3].

The width of the spectral curves (σ; [S3]) of the dyes was determined from the value of moment M_2:

$$\sigma = \sqrt{M_2},$$ (S4)

The asymmetry of the spectral curves and their shape [S3] are characterized by the non-dimensional γ-coefficients:

$$\gamma_1 = \frac{M_3}{\sigma^3},$$ (S5)

$$\gamma_2 = \frac{M_4}{\sigma^4} - 3$$ (S6)

4. Förster Resonance Energy Transfer

The parameters of Förster resonance energy transfer (FRET) between albumin donor chromophores (tyrosine and tryptophan amino acid residues of HSA) and acceptor (MCD **1**–**4** molecules) were determined from the spectral-fluorescent data using the standard approach [S2]. The spectral overlap integral (J, M^{-1} cm^{-1} nm^4) was calculated as

$$J(\lambda) \equiv \int_0^\infty \varepsilon_{Abs.}(\lambda)\lambda^4 F_D(\lambda)d\lambda,$$ (S7)

where ε(λ) is the molar absorption coefficient of the acceptor, L mol^{-1} cm^{-1}; $F(\lambda)$ is the normalized fluorescence spectrum of the donor; and λ is the wavelength, nm.
The critical radius of energy transfer (R_0) was determined from

$$R_0 = 0.211\left[\frac{\kappa^2\Phi_{f0}J(\lambda)}{n^4}\right]^{\frac{1}{6}},$$ (S8)

where κ^2 is the orientation factor, which depends on mutual orientation of the transition moments of the donor and the acceptor; Φ_{f0} is the fluorescence quantum yield for the donor in the absence of the acceptor; n is the refractive index of the medium; and λ is the wavelength, nm. In the calculations of R_0 by Equation S5, the value of the orientation factor κ^2 = 2/3 was used [S2], which corresponded to the random orientation of donor and acceptor molecules.

5. Determination of Binding Constants of the Dyes with DNA

To estimate the effective complexation constants (K_{eff}) of MCD **1**–**4** with biomacromolecules (BM) from fluorescence data constants, we used the Benesi–Hildebrand (K_{effBH} [S4]), Hill ($K_{effHill}$ [S5,S6]), and Scatchard (K_{1Sc} and K_{2Sc}) [S7, S8] formalizations.
If we consider the complexation of MCD1 – 4 with nucleic acids as a simple equilibrium of free (M_f) and bound dye monomers (M_b–BM)

$$M_f + BM_f \overset{K_{eff}}{\rightleftarrows} M_b\text{-}BM,$$ (S9)

then, to estimate the effective equilibrium constant (K_{eff}) of the complexation reaction of the dyes with DNA from the fluorescence data, we may use the equation of Benesi–Hildebrand (Equation S10; K_{effBH}, L mol^{-1}) applied to fluorescence techniques [S4]):

$$F = F_{max}\frac{K_{effBH}[BM_0]}{1+K_{effBH}[BM_0]},$$ (S10)

где $[BM_0] = c_{0BM}/n$ is the concentration of biomacromolecules (dsDNA, ssDNA, HSA) per one binding site, F is the fluorescence intensity of dyes in the presence of biomacromolecules, F_{max} is the fluorescence intensity at $[BM_0] \rightarrow \infty$, the ratio $K_{effBH}[BM_0]/(1 + K_{effBH}[BM_0])$ is the portion of dye molecules bound to the biopolymer. In coordinates $1/F$ vs. $1/[BM_0]$ dependence (S10) is a straight line with a slope of $1/(F_{max}$ $K_{effBH})$.

The Hill equation in the generally accepted form is as follows [S5,S6]:

$$\theta = \frac{F - F_0}{F_{\max} - F_0} = \frac{1}{\left(\frac{K_H}{BM_f}\right)^{n_{Hill}} + 1}, \quad \text{(S11)}$$

where F, F_0 are the fluorescence intensities of the dyes in the presence and in the absence of biomacromolecules (dsDNA, ssDNA, HSA), respectively; F_{max} can be obtained from Equation (S10), BM_f is the concentration of unbound biomacromolecules (under the considered conditions, we may assume $BM_f \approx c_{0BM}$ in base pairs); K_H is the Hill constant ($K_H = 1/K_{eff\mathrm{Hill}}$); and n_{Hill} is the Hill coefficient characterizing the binding cooperativity.

From Equation (S9), it is also possible to obtain a dependence similar to the Scatchard equation [S7,S8]:

$$\frac{[M_b - BM]}{[M_f]} = \frac{[M_b - BM]}{c_{0dye} - [M_b - BM]} \approx K_a \frac{c_{0BM}}{n_{bp}}, \quad \text{(S12)}$$

where $c_{0\mathrm{dye}}$ and $c_{0\mathrm{BM}}$ are the total concentrations of the dye and biomacromolecules, respectively, n_{bp} is the stoichiometric coefficient (for dsDNA it represents the number of DNA base pairs per one binding site of the MCD molecule).

The ratio $M_b\text{-}BM/(c_{0\mathrm{dye}} - M_b\text{-}BM)$ can be obtained from the fluorescence spectra:

$$M_b\text{-}BM/(c_{0\mathrm{dye}} - M_b\text{-}BM) = (F - F_0)/(F_{max} - F), \quad \text{(S13)}$$

Detection Limits

The limits of detection (LOD, μmol L^{-1}, μg mL^{-1}) and quantitative estimates (LOQ μmol L^{-1}, μg mL^{-1}) of biomacromolecules using MCD **1**–**4** as fluorescent probes were determined from the MCD fluorescence intensities using the following relationships:

$$\mathrm{LOD} = 3.3\sigma/k, \quad \text{(S14)}$$

$$\mathrm{LOQ} = 10\sigma/k = 3.3\mathrm{LOD}, \quad \text{(S15)}$$

where σ is the standard deviation (determined in the region of the smallest analyte concentrations detected), k is the slope of the linear range of the calibration curve (obtained from the initial points of the dependence of the dye fluorescence intensity on the albumin concentration) [S9].

Table S1. Solvatochromic properties of MCD **3** and **4** in some organic solvents: wavenumbers of the first moments of the normalized photon distribution and position of the band (ν_1, μm^{-1}; $M^{-1} = 10^7/\nu_1$, nm), spectral curve width (σ, cm^{-1}) of the absorption bands together with some properties of the media: dielectric constant (ε_r), refractive index (n), polarizability (P^*), Dimroth–Reichardt's $E_T(30)$.

					MCD 3			MCD 4		
**Solvent**	**ε_r**	***n***	***P****	**E_T(30)**	**ν_1, μm^{-1}**	**M^{-1}, nm**	**σ, 10^3 cm^{-1}**	**ν_1, μm^{-1}**	**M^{-1}, nm**	**σ, 10^3 cm^{-1}**
Acetonitrile	36.20	1.3441	0.2119	45.6	2.4261	412	1.214	1.9738	507	1.108
Acetone	20.70	1.3586	0.2199	42.2	2.4032	416	1.173	1.983	504	1.26
Ethanol	24.30	1.3614	0.2215	51.9	2.4108	415	1.127	1.9909	502	1.266
Ethyl acetate	6.02	1.3724	0.2275	38.1	2.3962	417	1.286	1.9716	507	1.271
1.4-Dioxane	2.22	1.4224	0.2464	36	2.3845	419	1.086	1.9606	510	1.300
Chloroform	4.81	1.4459	0.2586	39.1	2.3827	420	1.182	1.9562	511	1.183
DMSO	49.0	1.4793	0.2666	45.1	2.3941	418	1.175	1.9613	510	1.257

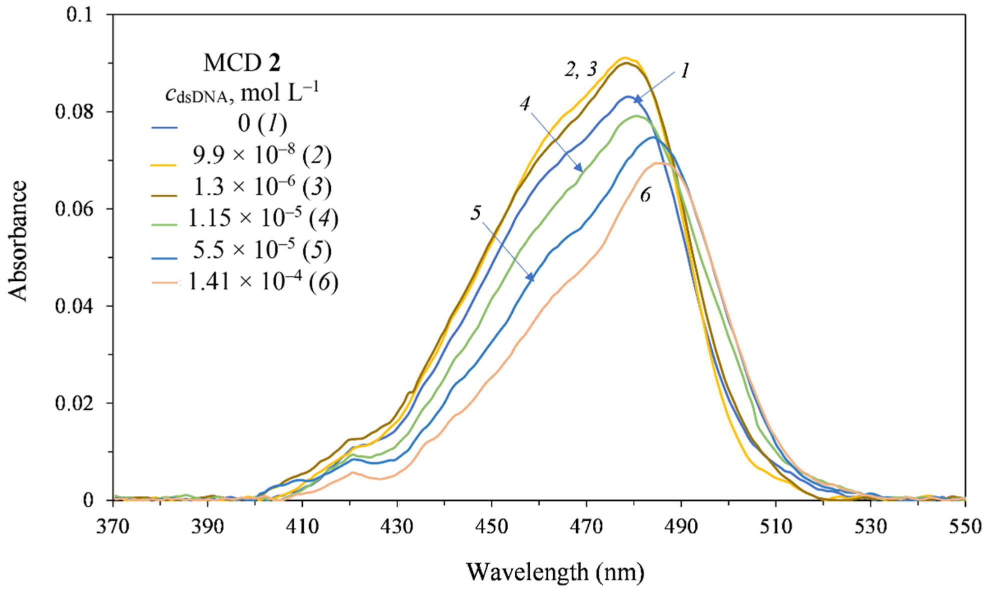

Figure S1. Absorption spectra of MCD **2** (c_{MCD2} ~ 1.2 × 10^{-6} mol L^{-1}) in PBS buffer in the presence of dsDNA: 0 (*1*), 9.9 × 10^{-8} (*2*), 1.3 × 10^{-6} (*3*), 1.15 × 10^{-5} (*4*), 5.5 × 10^{-5} (*5*) and 1.41 × 10^{-4} mol L^{-1} (*6*) dsDNA.

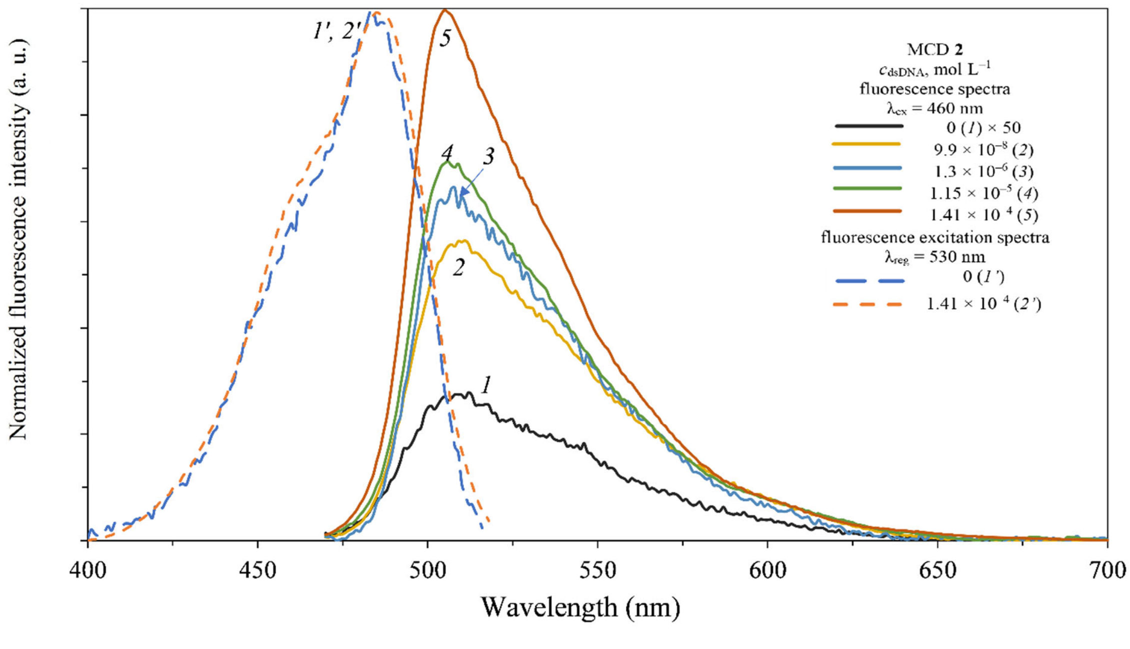

Figure S2. Normalized fluorescence (*1*–*5*; λ_{ex} = 460 nm) and fluorescence excitation (*1′*, *2′*; λ_{reg} = 530 nm) spectra of MCD **2** (c_{MCD2} ~ 1.2 × 10^{-6} mol L^{-1}) in PBS buffer in the presence of dsDNA: 0 (*1*, *1′*), 9.9 × 10^{-8} (*2*), 1.3 × 10^{-6} (*3*), 1.15 × 10^{-5} (*4*, *2′*), and 1.41 × 10^{-4} mol L^{-1} (*5*) dsDNA.

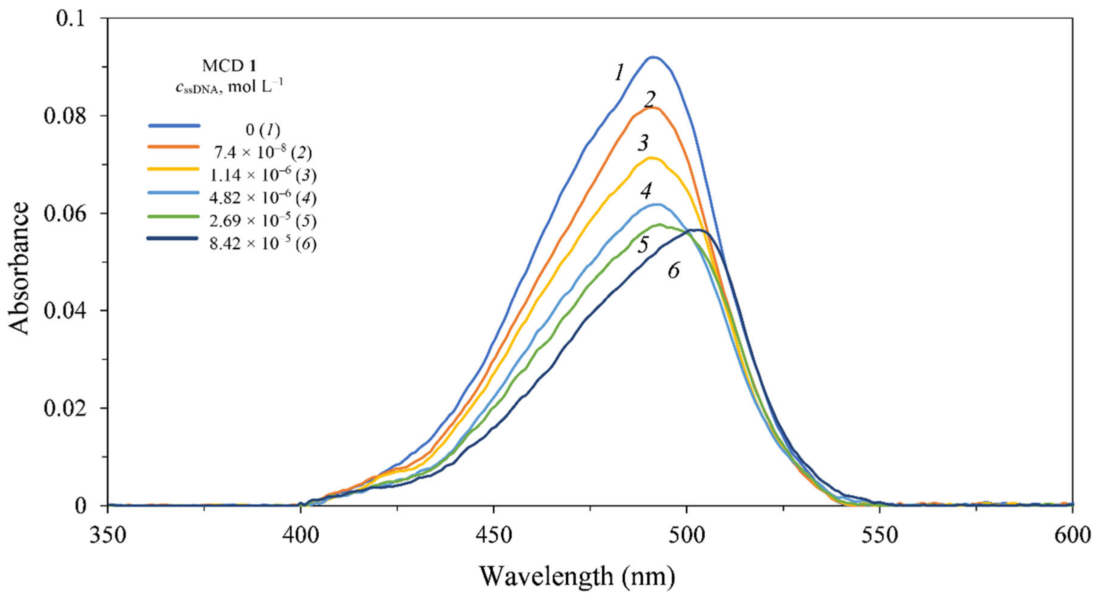

Figure S3. Absorption spectra of MCD **1** (c_{MCD1} = 2.25 × 10^{-6} mol L^{-1}) in PBS buffer in the presence of ssDNA: 0 (*1*), 7.4 × 10^{-8} (*2*), 1.14 × 10^{-6} (*3*), 4.82 × 10^{-6} (*4*), 2.69 × 10^{-5} (*5*) and 8.42 × 10^{-5} mol L^{-1} (*6*) ssDNA.

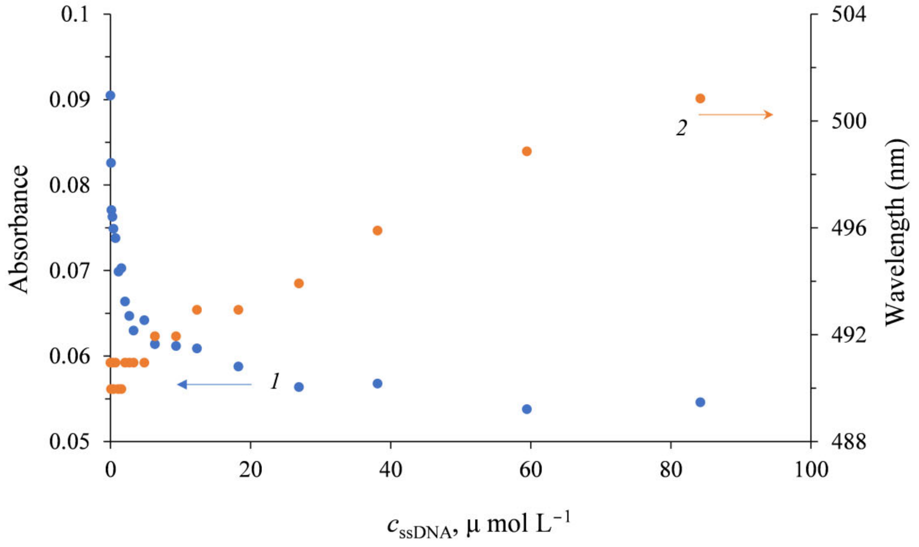

Figure S4. Plots of the MCD **1** absorbance (*1*) and position of the absorption spectral maximum (λ_{abs}^{max}, nm; *2*) vs. ssDNA concentration.

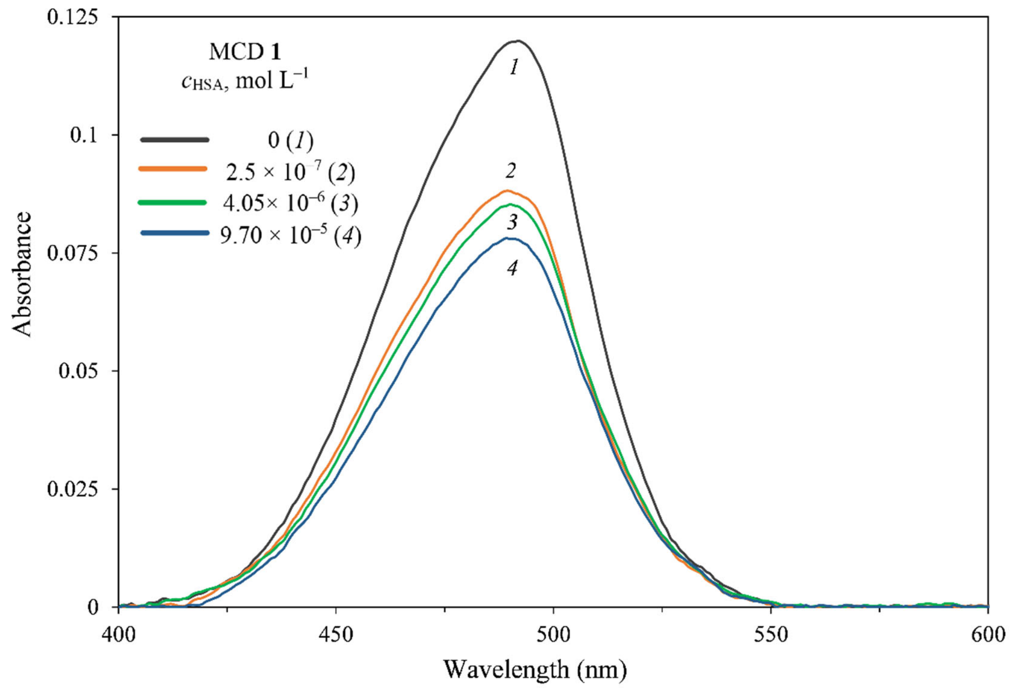

Figure S5. Absorption spectra of MCD **1** (c_{MCD1} ~ 3.0 × 10^{-6} mol L^{-1}) in PBS buffer in the presence of HSA: 0 (*1*), 2.5 × 10^{-7} (*2*), 4.05 × 10^{-6} (*3*), 9.7 × 10^{-5} (*4*) mol L^{-1} HSA.

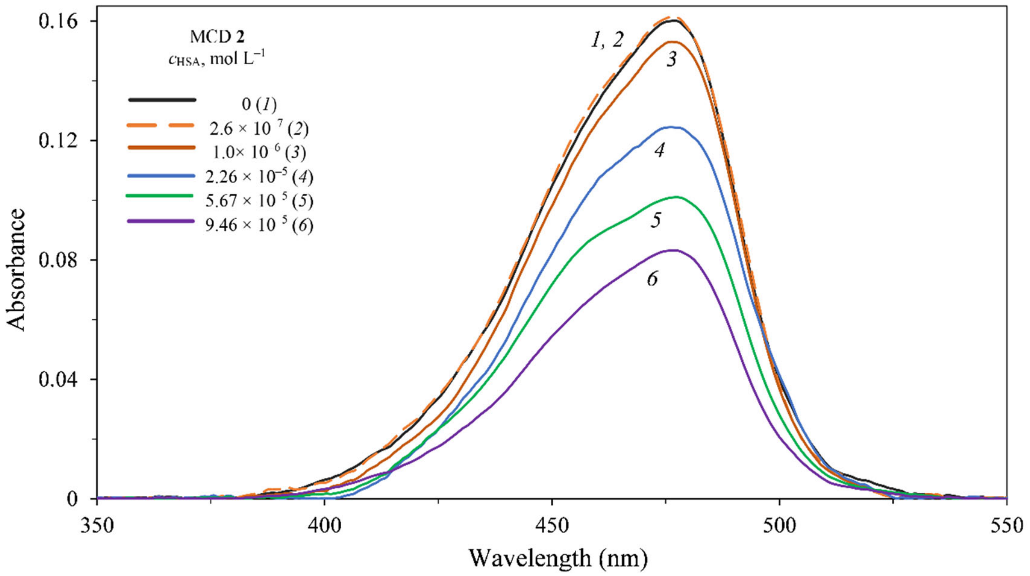

Figure S6. Absorption spectra of MCD **2** (c_{MCD2} ~ 2.4 × 10^{-6} mol L^{-1}) in PBS buffer in the presence of HSA: 0 (*1*), 2.6 × 10^{-7} (*2*), 1.00 × 10^{-6} (*3*), 2.26 × 10^{-5} (*4*), 5.67 × 10^{-5} (*5*), 9.46 × 10^{-5} (*6*) mol L^{-1} HSA.

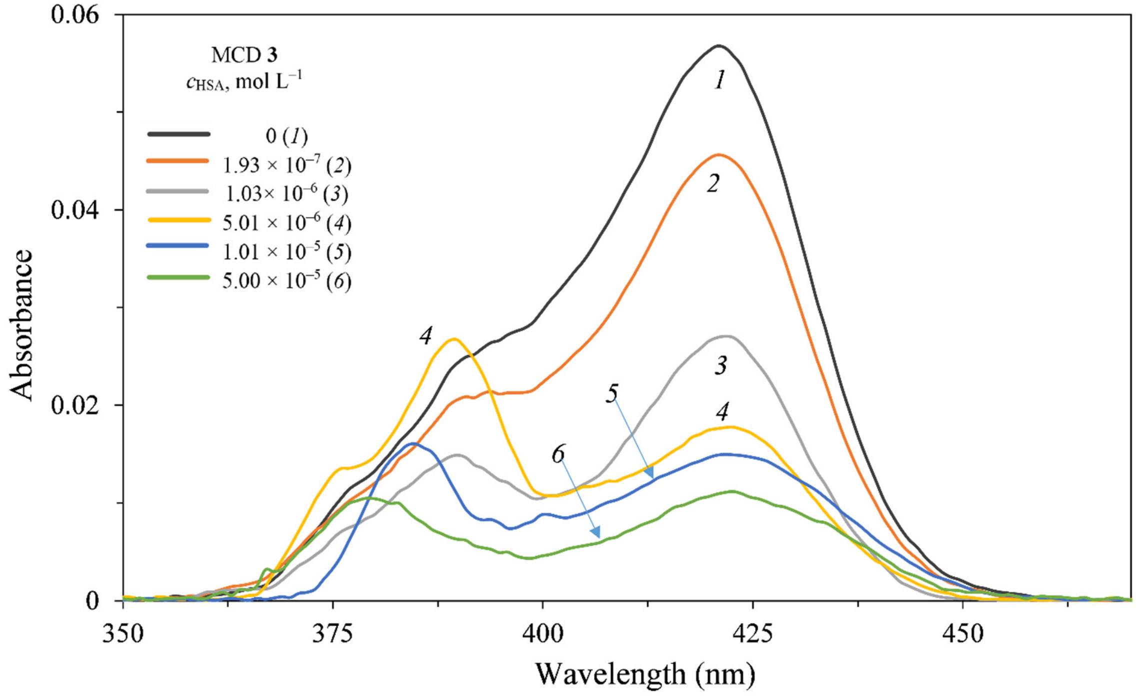

Figure S7. Absorption spectra of MCD **3** (c_{MCD3} ~ 1.0 × 10^{-6} mol L^{-1}) in PBS buffer in the presence of HSA: 0 (*1*), 1.93 × 10^{-7} (*2*), 1.03 × 10^{-6} (*3*), 5.01 × 10^{-6} (*4*), 1.01 × 10^{-5} (*5*), 5.0 × 10^{-5} (*6*) mol L^{-1} HSA.

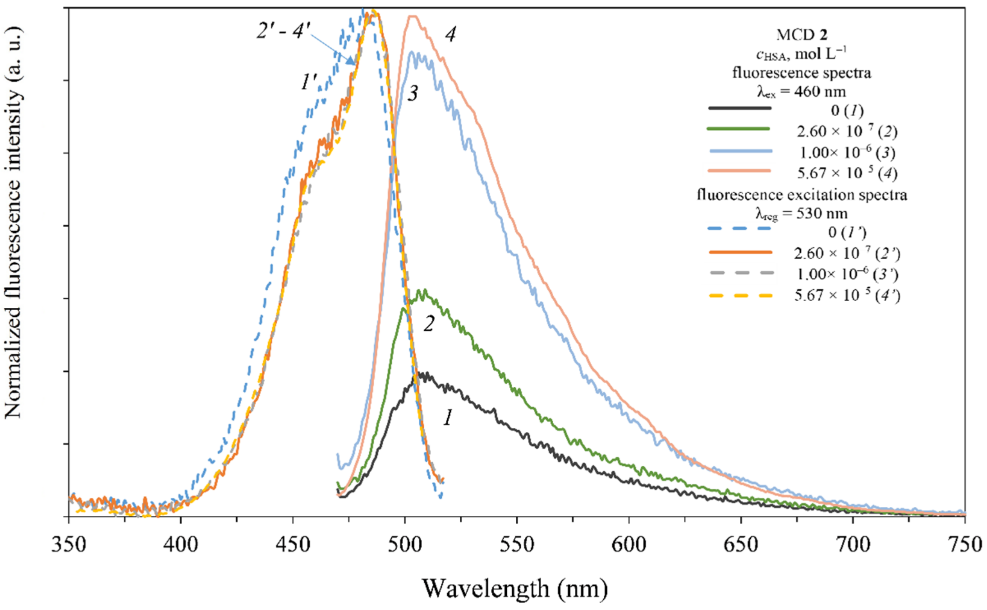

Figure S8. Normalized fluorescence (*1*–*4*; λ_{ex} = 460 nm) and fluorescence excitation spectra (*1′*– *4′*; λ_{reg} = 530 nm) of MCD **2** (c_{MCD2} ~ 2.4 × 10^{-6} mol L^{-1}) in PBS buffer in the presence of HSA: 0 (*1, 1′*), 2.6 × 10^{-7} (*2, 2′*), 1.00 × 10^{-6} (*3, 3′*), 5.67 × 10^{-5} (*4, 4′*) mol L^{-1} HSA.

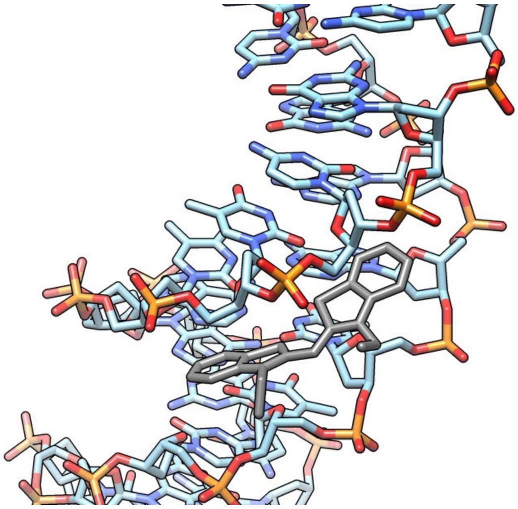

Figure S9. Results of molecular docking of MCD **3** with dsDNA (in the minor groove of dsDNA; 1BNA). Molecular graphics was performed with UCSF Chimera [S10].

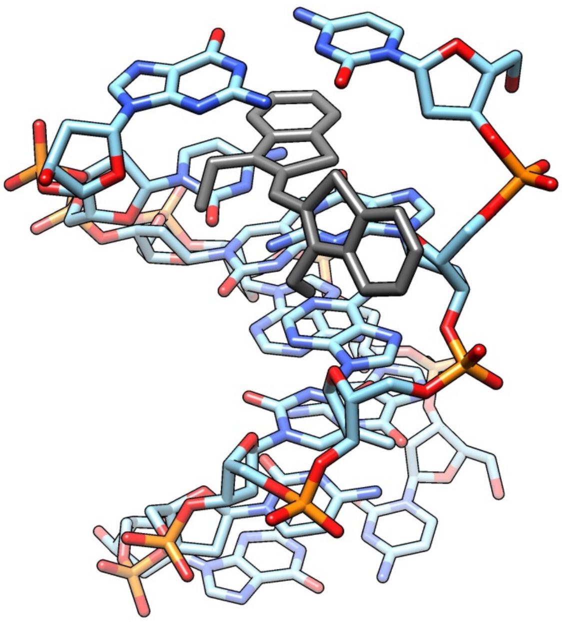

Figure S10. Results of molecular docking of MCD **3** with dsDNA in the half-intercalation mode (198D). Molecular graphics was performed with UCSF Chimera [S10].

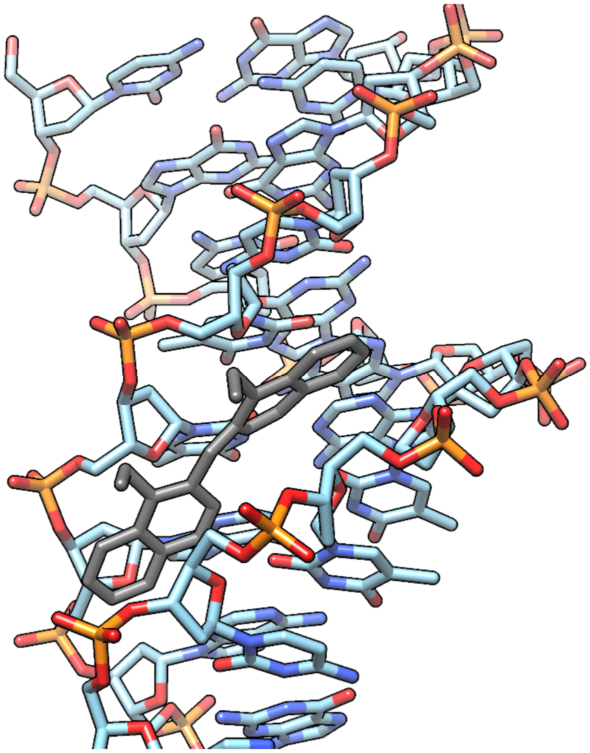

Figure S11. Results of molecular docking of MCD **4** with dsDNA (in the minor groove of dsDNA; 1BNA). Molecular graphics was performed with UCSF Chimera [S10].

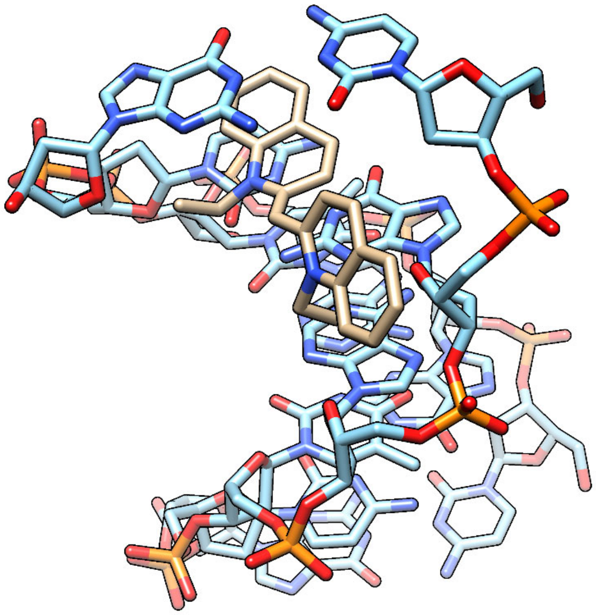

Figure S12. Results of molecular docking of MCD **4** with dsDNA in the half-intercalation mode (198D). Molecular graphics was performed with UCSF Chimera [S10].

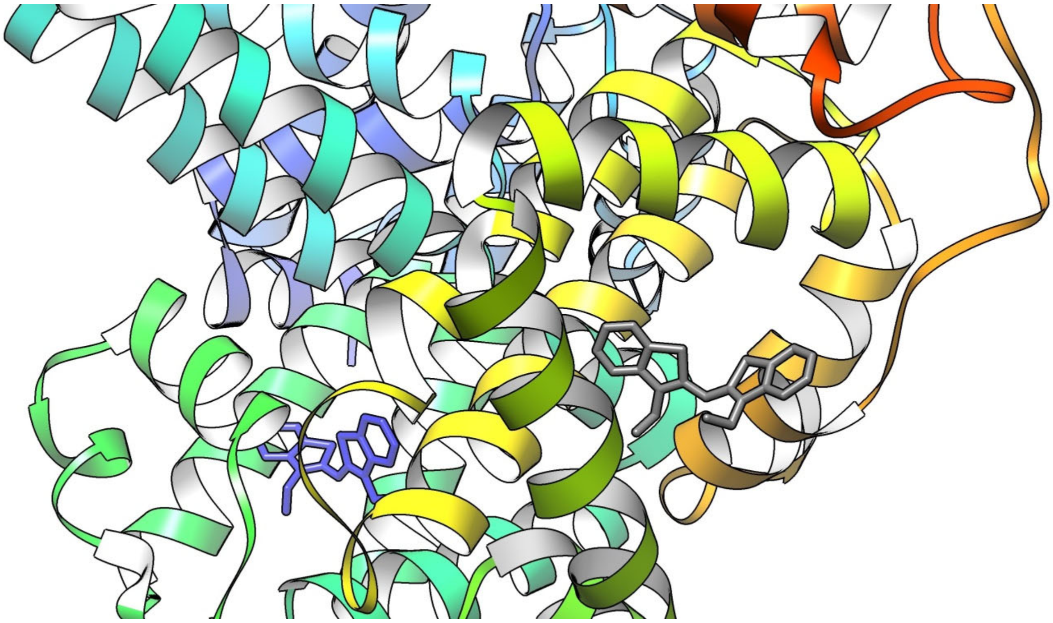

Figure S13. Results of molecular docking of MCD **3** with HSA (4K2C) Sidlow's site I (left, blue) and site II (right, gray). Molecular graphics was performed with UCSF Chimera [S10].

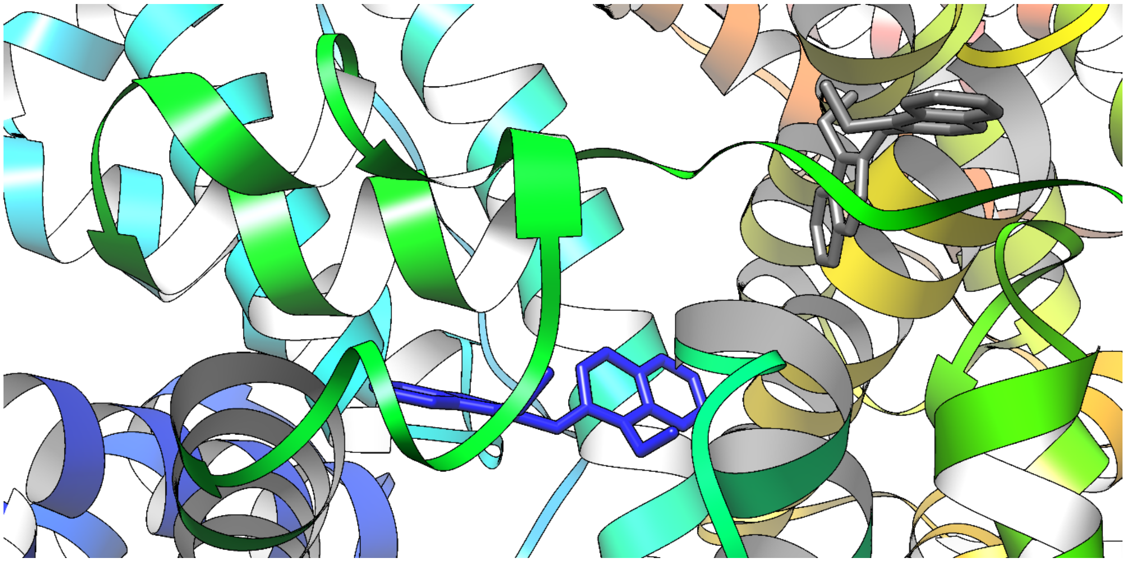

Figure S14. Results of molecular docking of MCD **4** with HSA (4K2C) Sidlow's site I (left, blue) and site II (right, gray). Molecular graphics was performed with UCSF Chimera [S10].

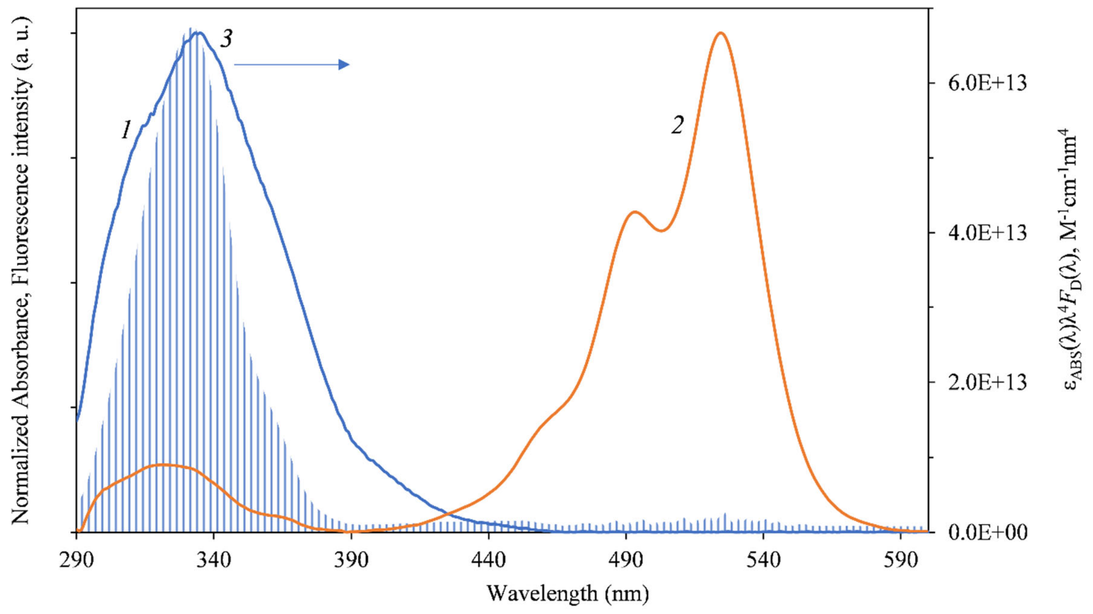

Figure S15. Overlapping of the fluorescence spectrum of HSA as a FRET donor (*1*, at λ_{ex} = 295 nm) and the absorption spectrum of MCD **4** as a FRET acceptor (*2*) in the UV region. The overlap integral of the donor and acceptor spectra is shown by the shaded area (*3*, right axis).

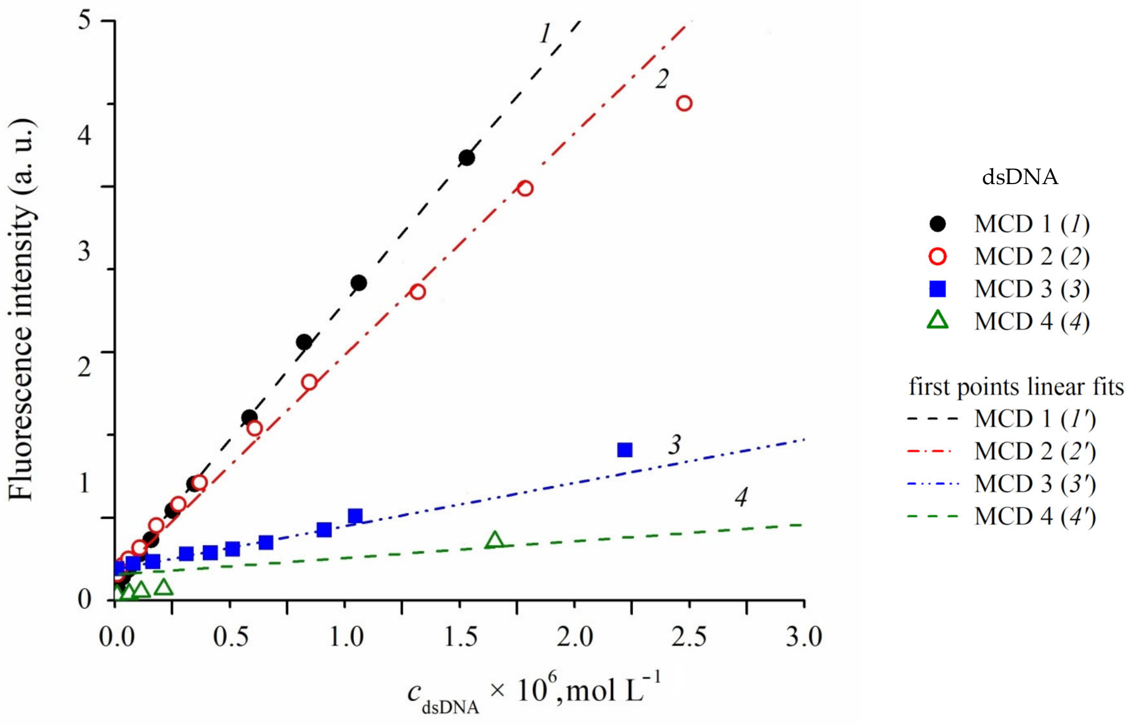

Figure S16. Plots of MCD **1** (*1*), **2** (*2*), **3** (*3*) and **4** (*4*) fluorescence intensity vs. dsDNA concentration (at low dsDNA concentrations).

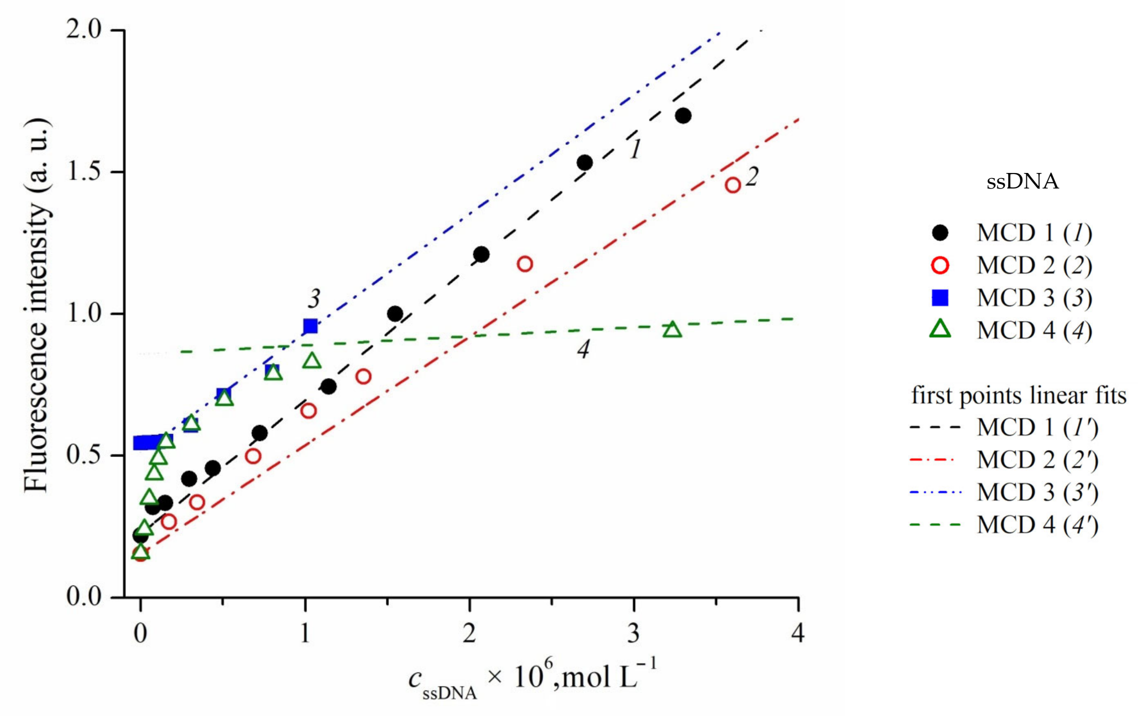

Figure S17. Plots of MCD **1** (*1*), **2** (*2*), **3** (*3*) and **4** (*4*) fluorescence intensity vs. ssDNA concentration (at low ssDNA concentrations).

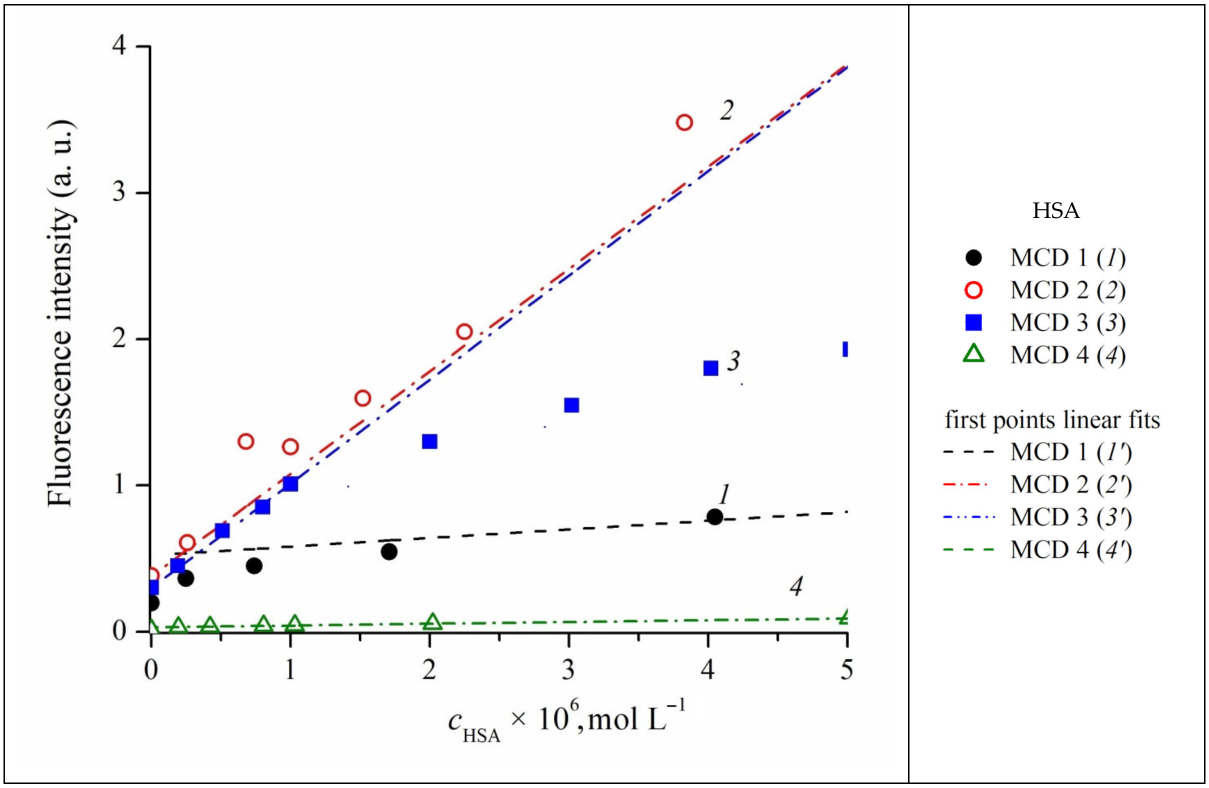

Figure S18. Plots of MCD **1** (*1*), **2** (*2*), **3** (*3*) and **4** (*4*) fluorescence intensity vs. HSA concentration (at low HSA concentrations).

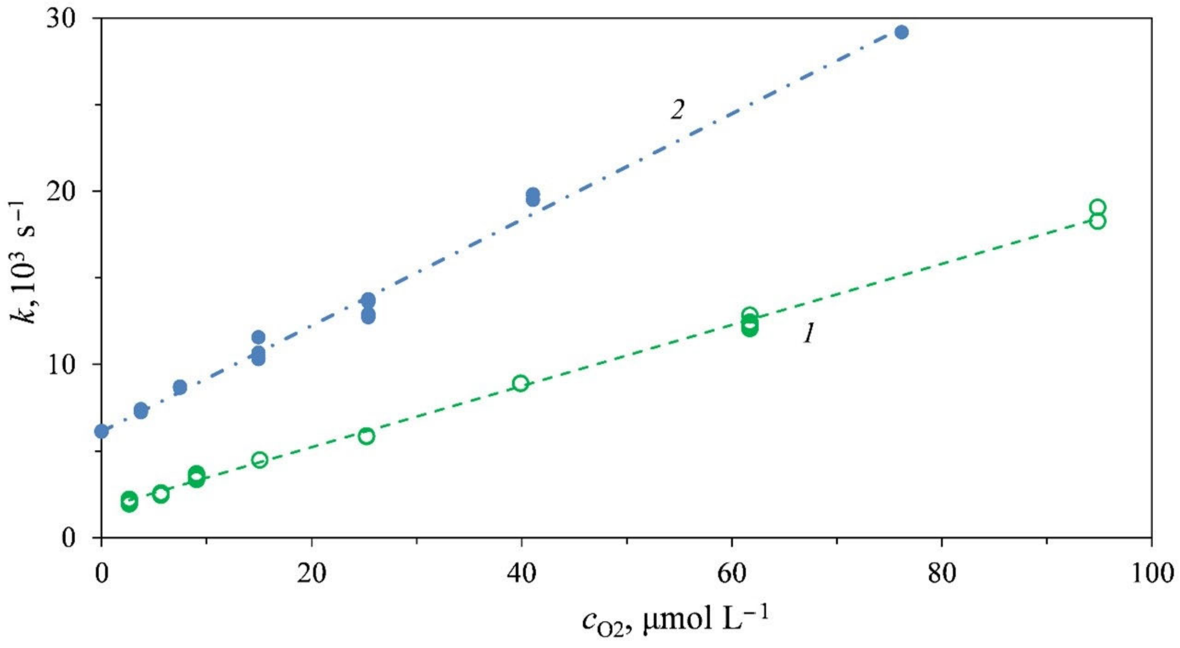

Figure S19. Dependences of the triplet state decay rate constants (k, 10^3 s^{-1}) of MCD **2** (*1*, λreg = 600 nm) and **3** (*2*, λ_{reg} = 650 nm), on the concentration of dissolved oxygen in the presence of dsDNA (c_{dsDNA} = 1.0 × 10^{-4} mol L^{-1}).

References

S1. Demas, J.N.; Crosby, G.A. Measurement of photoluminescence quantum yields. Review, *J. Phys. Chem.* **1971**, *75*, 991–1024. https://doi.org/10.1021/j100678a001.

S2. Lakowicz, J.R. Principles of Fluorescence Spectroscopy, 3rd edition Springer ISBN: 978-0-387-31278-1 Hardcover, 2006, 954 p.

S3. Dyadyusha, G.G.; Ishchenko, A.A. Application of the method of moments to the study of the electronic spectra of organic dyes, *J. Appl. Spectrosc.* **1979**, *30*, 746–750. https://doi.org/10.1007/BF00615763

S4. Benesi, H.; Hildebrand, J. A spectrophotometric investigation of the interaction of iodine with aromatic hydrocarbons, *J. Am. Chem. Soc.* **1949**, *71*, 2703–2707, https://doi.org/10.1021/ja01176a030.2.

S5. Hill, A.V. The possible effects of the aggregation of the molecules of haemoglobin on its dissociation curves, *J. Physiol.* **1910**, *40*, iv-vii.

S6. Gesztelyi, R.; Zsuga, J.; Kemeny-Beke, A.; Varga, B.; Juhasz, B.; Tosaki, A. The Hill equation and the origin of quantitative pharmacology, *Arch. Hist. Exact Sci.* **2012**, *66*, 427–438. https://doi.org/10.1007/s00407-012-0098-5.

S7. Scatchard, G. The attractions of proteins for small molecules and ions, *Ann. N.Y. Acad. sci.* **1949**, *51*, 660–672. https://doi.org/10.1111/j.1749-6632.1949.tb27297.x.

S8. Nørby, J.G.; Ottolenghi, P.; Jensen, J. Scatchard plot: Common misinterpretation of binding experiments, *Anal. Biochem.* **1980**, *102*, 318–320. https://doi.org/10.1016/0003-2697(80)90160-8.

S9. MacDougall, D.; Crummett, W.B. et al., Guidelines for data acquisition and data quality evaluation in environmental chemistry, *Anal. Chem.* **1980**, *52*, 2242–2249. https://doi.org/10.1021/ac50064a004.

S10. Pettersen, E.F.; Goddard, T.D.; Huang, C.C.; Couch, G.S.; Greenblatt. D.M.; Meng, E.C.; Ferrin. T.E. UCSF Chimera—A visualization system for exploratory research and analysis. *J. Comput. Chem.* **2004**, *25(13)*, 1605–1612. https://doi.org/10.1002/jcc.20084.
